# Psychological–dietary interactions in chronic disease management: mechanisms and integrated intervention strategies

**DOI:** 10.3389/fnut.2026.1925415

**Published:** 2026-09-09

**Authors:** Xiunan Liu, Hongwei Leng, Zhuoxing Li, Xumao Deng, Buyuan Tian, Xiang Xiao, Yun Sun

**Affiliations:** 1 School of Clinical Medicine, Chengdu Medical College, Chengdu, China; 2 Department of Nephrology, The First Affiliated Hospital of Chengdu Medical College, Chengdu, China

**Keywords:** chronic disease, dietary adherence, dietary behavior, psychological distress, self-management

## Abstract

**Objective:**

This narrative review aims to summarize evidence from nutritional psychiatry, behavioral medicine, and chronic disease management, examine the relationship between psychological factors and dietary behaviors in chronic disease management, and discuss potential mechanisms, disease-specific manifestations, and clinical management approaches.

**Background:**

Chronic disease management involves not only the control of disease-related indicators but also the long-term maintenance of dietary behaviors, treatment adherence, and self-management. Stress, anxiety, depression, reduced self-efficacy, and disease-related distress may affect food choices, eating behaviors, and dietary adherence, while dietary patterns, nutrient intake, and dietary restriction may also be associated with emotional and cognitive status. Therefore, psychological factors and dietary behaviors may influence each other during long-term chronic disease management.

**Results:**

Existing evidence suggests that psychological factors and dietary behaviors may be bidirectionally associated. Psychological distress may be related to emotional eating, preference for energy-dense foods, excessive dietary restriction, and reduced dietary adherence, while dietary behaviors may also be associated with psychological status and chronic disease management through pathways involving the hypothalamic–pituitary–adrenal (HPA) axis, reward processing, inflammation, the gut-brain axis, and metabolic changes. These relationships may differ across chronic diseases, with obesity, type 2 diabetes mellitus (T2DM), cardiovascular disease (CVD), chronic gastrointestinal diseases, and chronic kidney disease (CKD) presenting different psychological and dietary management challenges. Psychological screening, individualized nutritional guidance, behavioral support, mindful eating, digital monitoring, and multidisciplinary collaboration may provide additional support for long-term chronic disease management.

**Conclusion:**

Existing evidence suggests a potential bidirectional relationship between psychological factors and dietary behaviors in chronic disease management. Based on this evidence, this review further proposes an integrated psychological–dietary management framework, although its overall clinical effectiveness and specific implementation approaches still require further evaluation. More prospective studies, randomized controlled trials (RCTs), and real-world implementation studies are needed before this approach can be further developed for routine or standardized chronic disease management.

## 1 Introduction

With global population aging, accelerated urbanization, and changes in lifestyle and dietary patterns, noncommunicable diseases (NCDs), such as obesity, type 2 diabetes mellitus (T2DM), cardiovascular disease (CVD), chronic gastrointestinal diseases, and chronic kidney disease (CKD), have become major public health challenges worldwide. Data from the World Health Organization (WHO) showed that NCDs caused at least 43 million deaths globally in 2021, accounting for approximately 75% of all non-pandemic-related deaths worldwide (1). The Global Burden of Disease (GBD) Study also indicated that NCDs remain an important contributor to the global burden of mortality and disability (2). In China, the burden of chronic diseases is similarly substantial. In 2021, NCDs accounted for 91.0% of total deaths and 86.7% of total disability-adjusted life-years (DALYs) (3). Because chronic diseases are characterized by long disease courses, prolonged treatment periods, and a strong reliance on patient self-management, their impact is reflected not only in mortality and disability burden, but also in reduced quality of life, increased treatment burden, and greater healthcare resource consumption (4). Therefore, strategies for the prevention and management of chronic diseases urgently need to shift from simple disease control toward more systematic, continuous, and patient-centered integrated management.

Dietary behaviors and psychological factors are two important and interrelated dimensions in the prevention, control, and long-term management of chronic diseases. Unhealthy diets are important and modifiable risk factors contributing to the global disease burden. The GBD Study showed that, in 2017, approximately 11 million deaths and 255 million DALYs worldwide were attributable to dietary risks (5). At the same time, chronic disease management is not merely a process of dietary modification or disease indicator control, but also a process of maintaining long-term behaviors. Patients need to continuously adjust their dietary patterns, control body weight, follow treatment regimens, and monitor disease status, and this process is often accompanied by psychological stress, disease-related distress, and difficulties with adherence. The WHO report on adherence to long-term therapies noted that poor adherence to long-term treatment for chronic diseases is a global problem, with average adherence to long-term therapy in developed countries being approximately 50% (6). Previous studies have also suggested that psychological distress and related behavioral factors may be associated with poorer self-management and treatment outcomes (7–10). Therefore, difficulties in maintaining dietary behaviors and reduced adherence among patients with chronic diseases should not be simply attributed to a lack of willpower, but should be understood in the context of psychological status, disease burden, and long-term self-management capacity.

Current chronic disease management is gradually shifting from single-disease treatment toward multidisciplinary integrated management, with nutritional guidance, behavioral interventions, and psychological support increasingly incorporated into long-term care. However, in clinical practice, dietary management and psychological support often remain relatively separate. Dietary interventions tend to focus on nutrient intake, energy balance, and metabolic indicators, whereas psychological interventions mainly address emotional symptoms, stress, and psychological adjustment, with limited integration between the two. Existing studies suggest that psychological states such as stress, depression, and anxiety may influence food choices and dietary control (11, 12), while dietary patterns, nutrient intake, and metabolic status may also be associated with mood and cognitive function (13, 14). Therefore, interventions focusing only on diet or psychological factors may not fully address the difficulties that patients with chronic diseases face in maintaining long-term behavioral changes and managing their conditions.

**Figure 1:**
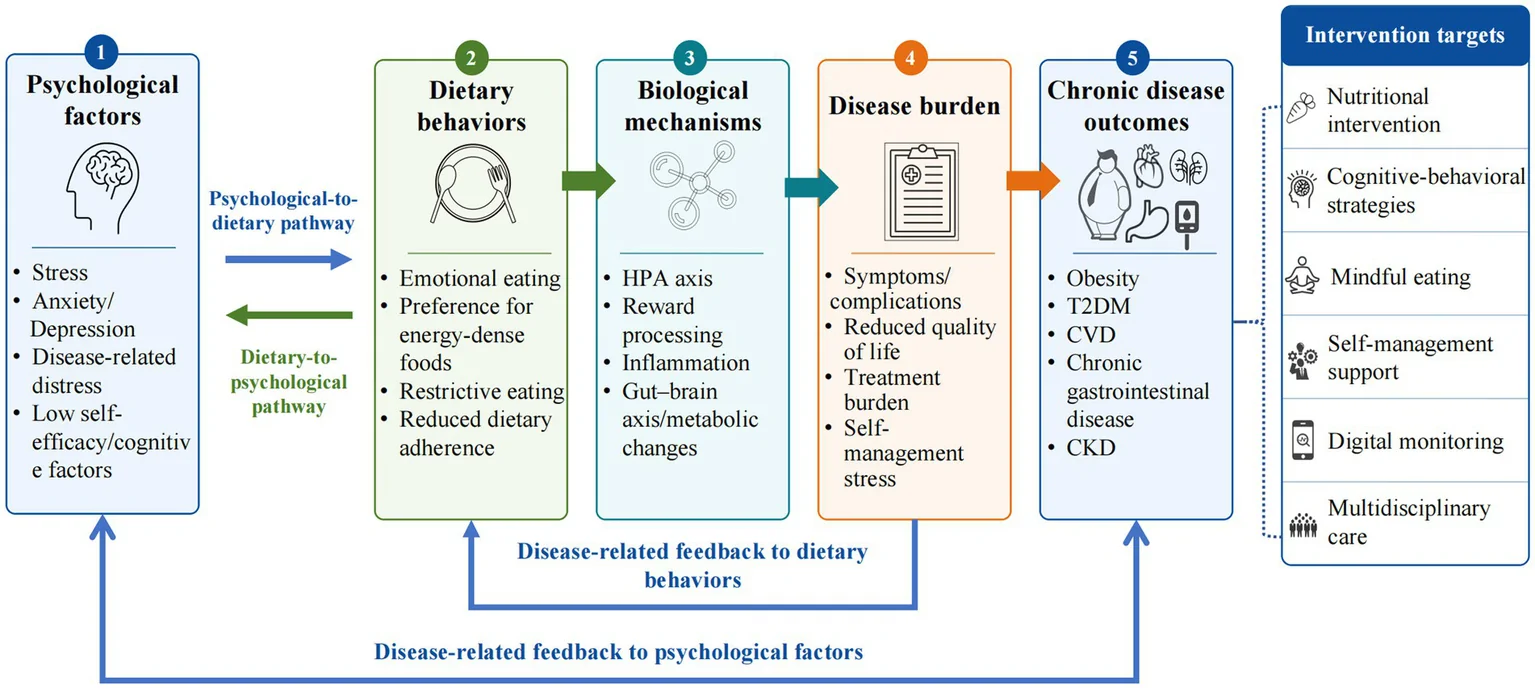
Conceptual framework linking psychological factors and dietary behaviors in chronic disease management. This framework illustrates the potential bidirectional and dynamic relationships among psychological factors, dietary behaviors, biological mechanisms, disease burden, and chronic disease outcomes. Psychological factors, including stress, anxiety/depression, disease-related distress, and low self-efficacy or cognitive factors, may influence dietary behaviors such as emotional eating, preference for energy-dense foods, restrictive eating, and reduced dietary adherence. Conversely, dietary behaviors may also influence psychological status, forming a bidirectional relationship between psychological and dietary factors. These relationships may involve biological mechanisms such as the hypothalamic-pituitary-adrenal (HPA) axis, reward processing, inflammation, and the gut–brain axis or metabolic changes. Disease burden, including symptoms or complications, reduced quality of life, treatment burden, and self-management stress, may further feed back on psychological status and dietary behaviors and may be linked to chronic disease outcomes such as obesity, type 2 diabetes mellitus, cardiovascular disease, chronic gastrointestinal diseases, and chronic kidney disease. The framework also summarizes potential intervention targets, including nutritional intervention, cognitive-behavioral strategies, mindful eating, self-management support, digital monitoring, and multidisciplinary care. Overall, the figure highlights the ongoing mutual influence between psychological factors and dietary behaviors during chronic disease management, together with the dynamic feedback from disease burden and disease outcomes. HPA, hypothalamic-pituitary-adrenal; T2DM, type 2 diabetes mellitus; CVD, cardiovascular disease; CKD, chronic kidney disease.

This narrative review aims to discuss the dynamic interactions between psychological factors and dietary behaviors in chronic disease management, summarize their potential mechanisms, disease-specific manifestations, and clinical intervention strategies, and provide an analytical perspective for future research on integrated psychological–dietary management. It should be noted that the psychological–dietary interactions discussed in this review do not refer to a specific, validated neurobiological circuit. Rather, they represent an analytical perspective for understanding the interrelationships among psychological status, dietary behaviors, biological mechanisms, disease burden, and intervention targets in chronic disease management. Unlike nutritional psychiatry, which mainly focuses on the relationships among dietary patterns, nutrient intake, and mental health (15), this review places greater emphasis on the role of these relationships in long-term self-management, dietary adherence, and disease burden among patients with chronic diseases. Unlike the gut–brain axis, which primarily emphasizes biological pathways involving the gut microbiota, neuroendocrine regulation, immunity, and metabolism (16), this review also incorporates emotional, cognitive, behavioral, social environmental, and clinical implementation factors. Compared with the traditional biopsychosocial model (17), this review further focuses on the bidirectional feedback between psychological status and dietary behaviors, as well as its potential intervention value in chronic disease management. Figure 1 summarizes the proposed conceptual framework linking psychological factors and dietary behaviors in chronic disease management.

## 2 Methods

### 2.1 Literature search and study selection

As this was a narrative review, no systematic review registration was conducted and no PRISMA flow diagram was prepared. This review focused on the interactions between psychological factors and dietary behaviors in chronic disease management. Relevant literature was searched in PubMed, Web of Science, Embase, and Scopus from database inception to April 2026. Detailed database-specific search strategies are provided in Supplementary Table S1.

Studies addressing psychological factors, dietary behaviors, related biological mechanisms, and psychological or dietary management in obesity, type 2 diabetes mellitus, cardiovascular disease, chronic gastrointestinal diseases, and chronic kidney disease were considered for inclusion. Studies on nutritional, psychological, behavioral, digital, and integrated psychological–dietary interventions were also included based on their relevance to the topic of this review. Studies with limited relevance to the topic, duplicate content, incomplete study information, or unavailable full text were excluded.

### 2.2 Evidence synthesis and interpretation

Because the available studies differed in study design, study population, intervention approach, and outcome measures, the evidence was summarized and synthesized narratively without quantitative pooling. Systematic reviews and meta-analyses, randomized controlled trials, and longitudinal studies were used as the main sources of evidence. Cross-sectional and other observational studies were mainly used to describe associations among psychological factors, dietary behaviors, and outcomes related to chronic disease management. Mechanistic and experimental studies were used to help explain potential biological processes, while qualitative studies provided additional information on patient experiences, behavioral barriers, and clinical implementation. Table 1 summarizes the main evidence types and relevant references for each chronic disease section.

**Table 1:** Overview of psychological factors, dietary management challenges, potential mechanisms, and intervention strategies across major chronic diseases.

Chronic disease	Main psychological factors	Main dietary challenges	Potential mechanisms	Potential interventions	Main evidence types and representative references
Obesity (9, 88, 89, 91–93, 130)	Stress, anxiety/depression, psychological burden related to emotional eating, weight stigma, guilt after eating, and a sense of loss of control	Emotional eating, preference for high-fat, high-sugar, and energy-dense foods, loss-of-control eating, and difficulties with long-term weight management	Stress-related changes in the HPA axis and reward processing, as well as feedback among emotion, cognition, self-regulation, and eating behaviors	Individualized nutritional and behavioral support, greater awareness of eating behaviors, mindful eating, and avoidance of weight stigma as a strategy for behavior change	Experimental studies, narrative reviews, prospective/longitudinal observational studies, systematic reviews, and meta-analyses
T2DM (25, 94, 95, 98, 114, 132)	Diabetes distress, depressive symptoms, fear of hypoglycemia, dietary guilt, and frustration with self-management	Long-term dietary control, carbohydrate management, prevention of hypoglycemia, and dietary adherence	Interrelationships among diabetes distress, self-management behaviors, and glycemic control, together with pathways involving self-efficacy and behavioral adherence	Diabetes education, problem-solving, self-management support, more flexible dietary planning, and hypoglycemia risk management; further psychological assessment when needed	Guidelines/expert consensus, cross-sectional and longitudinal studies, systematic reviews, and meta-analyses
CVD (77, 78, 80, 99, 100, 104, 115, 122, 138, 139)	Chronic stress, anxiety, and depression	Preference for high-fat, high-sugar, and high-salt foods, and difficulties maintaining low-salt, healthy-fat, and cardioprotective dietary patterns over time	Inflammation, metabolic abnormalities, neuroendocrine responses, and the influence of psychological status on lifestyle behaviors and treatment adherence	Healthy dietary patterns such as the Mediterranean and DASH diets, psychological screening, behavioral support, and psychological intervention when needed	Reviews, randomized controlled trials, observational studies, professional society recommendations, systematic reviews, and meta-analyses
IBS/IBD (26, 74, 106, 108–110, 116)	Food-related anxiety, concerns about symptoms, anticipatory anxiety, and psychological burden related to restrictive eating	Food avoidance, excessive dietary restriction, burden of implementing a low-FODMAP diet, and potential inadequate nutrient intake	Behavioral feedback among symptoms, food attribution, anxiety, and avoidance, together with potential biological pathways such as the gut–brain axis	Professionally guided low-FODMAP diet, gradual food reintroduction, and individualized adjustment; avoidance of unnecessary long-term restriction and attention to marked restrictive eating behaviors	Clinical observational studies, evidence-based reviews, systematic scoping reviews, prospective multicenter observational studies, and systematic reviews
CKD (27, 112, 113, 117)	Dietary restriction fatigue, psychological stress, and the burden of chronic illness and long-term self-management	Complex restrictions involving protein, sodium, potassium, phosphorus, and fluid intake, together with difficulties maintaining long-term adherence	Social limitations, changes in eating habits, self-control burden, and treatment burden associated with long-term dietary restrictions may jointly affect dietary management	Individualized renal nutritional management according to kidney function, laboratory indicators, and dialysis status, together with identification of dietary restriction fatigue and substantial psychological burden, with additional support or referral when needed	Qualitative evidence syntheses, clinical practice guidelines, integrative reviews, and clinical nutrition reviews

HPA, hypothalamic–pituitary–adrenal; T2DM, type 2 diabetes mellitus; CVD, cardiovascular disease; IBS, irritable bowel syndrome; IBD, inflammatory bowel disease; CKD, chronic kidney disease; DASH, Dietary Approaches to Stop Hypertension; FODMAP, fermentable oligosaccharides, disaccharides, monosaccharides, and polyols.

## 3 Effects of psychological factors on dietary behaviors

Psychological factors may be associated with dietary adherence and long-term behavioral maintenance among patients with chronic diseases. For these patients, dietary behaviors are not only reflections of physiological needs, but are also influenced by emotional status, stress-coping styles, illness perceptions, self-efficacy, and the social environment. Stress, anxiety, depression, disease-related distress, and low self-efficacy may alter food choices, eating patterns, and dietary control, thereby affecting the effectiveness of chronic disease management. Therefore, understanding how psychological factors influence dietary behaviors may help explain why patients have difficulty adhering to dietary recommendations over the long term and may provide a basis for subsequent stratified interventions.

### 3.1 Individual intrinsic factors

#### 3.1.1 Emotion and stress

Emotion is one of the important psychological factors influencing dietary behaviors. Negative emotions, such as stress, anxiety, and depression, are often associated with emotional eating, in which individuals are more likely to choose high-sugar, high-fat, and high-energy foods to obtain temporary emotional relief or reward experiences (18). This process is particularly important among patients with chronic diseases, because long-term disease burden, treatment-related stress, and lifestyle restrictions may themselves become persistent sources of psychological stress and further affect patients’ dietary choices and adherence (19). However, the influence of emotion on dietary behaviors is not unidirectional. Positive emotions may promote healthy food choices and self-regulation, but may also enhance hedonic eating behaviors in certain contexts (20). Therefore, the relationship between emotion and diet should be understood in relation to individual self-regulatory capacity, reward sensitivity, and specific eating contexts.

The effects of stress on dietary behaviors are also complex. Acute stress may alter appetite, food reward responses, and tendencies toward energy intake over a short period through activation of the sympathetic nervous system and the hypothalamic–pituitary–adrenal (HPA) axis (21). Some individuals may experience increased appetite under stress, with a particular preference for high-sugar, high-fat, and high-energy foods, whereas others may experience reduced appetite or decreased food intake (22). In contrast, the effects of chronic stress are more persistent. Long-term disease burden and lifestyle restrictions may contribute to sustained activation of the HPA axis or dysregulation of stress responses, thereby affecting insulin sensitivity, inflammatory responses, satiety signaling, and related processes (23). In addition, chronic stress may weaken cognitive control and self-regulatory capacity, making patients more prone to impulsive eating, loss of dietary control, and reduced dietary adherence under conditions of fatigue, negative emotions, or disease-related stress (24).

#### 3.1.2 General psychological symptoms and disease-related distress

Among patients with chronic diseases, psychological factors that may influence dietary behaviors include not only general psychological symptoms such as stress, anxiety, and depression, but also disease-related distress associated with specific disease management tasks. General psychological symptoms may affect dietary behaviors by influencing behavioral motivation, self-efficacy, and dietary control, whereas disease-related distress more often arises from treatment requirements, dietary restrictions, concerns about complications, and the burden of long-term self-management, with its manifestations varying across diseases.

For example, patients with T2DM may experience diabetes distress (25), patients with chronic gastrointestinal diseases may develop food-related anxiety and dietary avoidance (26), and patients with CKD may experience dietary burden and dietary restriction fatigue (27). These problems are not entirely equivalent to general depression or anxiety, but are more closely related to the specific demands of disease management. When long-term management becomes difficult, patients may also experience guilt, self-blame, or a reduced sense of control, which may further affect subsequent dietary management.

Therefore, when assessing dietary behaviors in patients with chronic diseases, it is important to distinguish general psychological symptoms from disease-related distress. The two may coexist and jointly influence food choices, dietary control, and long-term behavioral maintenance. Incorporating disease-related distress into the psychological–dietary framework may help identify the psychological and behavioral barriers patients face in dietary management more accurately and provide a basis for subsequent interventions.

#### 3.1.3 Cognition, beliefs, and self-efficacy

In addition to emotion, stress, and disease-related distress, individual cognitive appraisal, illness beliefs, and self-efficacy also influence dietary behaviors. Cognitive factors do not simply refer to whether patients understand healthy eating knowledge, but also include how they interpret their disease, whether they believe they can adhere to dietary recommendations, and how they explain their own behavior after deviating from dietary plans.

Previous studies on eating disorders have suggested that self-esteem, body image, and excessive concerns about weight or body shape may affect patients’ dietary control behaviors (28). Low self-esteem and body dissatisfaction may make individuals more likely to adopt extreme dieting, excessive control, or compensatory eating behaviors, and may lead to stronger self-blame and shame after loss of dietary control (29). These studies provide important clues for understanding diet-related cognition and loss of behavioral control. However, among patients with chronic diseases, these cognitive mechanisms do not necessarily present as typical eating disorders, but are more often reflected in dietary concerns under disease management, feelings of dietary failure, resistance to dietary restrictions, worries about disease progression, and reduced self-efficacy. Therefore, in chronic disease management, diet-related cognitive problems should be understood in the context of disease self-management and long-term adherence, rather than being interpreted simply through the framework of eating disorders.

In addition, illness beliefs may affect how patients with chronic diseases accept and implement dietary recommendations. Taking T2DM as an example, a study including 893 participants showed that, compared with individuals without diabetes, patients with T2DM had lower scores for beliefs about the modifiability of blood glucose and for self-efficacy related to general health, blood glucose, and cholesterol. Among patients with T2DM, lower general health- and blood glucose-related growth mindsets or self-efficacy were associated with higher glycated hemoglobin (HbA1c) levels (30). This suggests that whether patients believe their disease indicators can be improved through behavioral changes may be related to their self-management status. Self-efficacy is also an important factor influencing the maintenance of dietary behaviors. Even when patients have strong intentions to eat healthily, they may find it difficult to adhere to dietary recommendations over the long term under stress, symptom fluctuations, social eating situations, or complex dietary rules if they lack confidence in their own ability to carry them out (31).

Therefore, cognition, beliefs, and self-efficacy are important mediators linking psychological status and dietary behaviors. Dietary interventions for chronic diseases should not be limited to telling patients “what they should eat”; they should also help patients understand their dietary goals and improve their confidence in dietary management through achievable small goals. Incorporating illness perceptions, self-efficacy, and perceived behavioral control into the psychological–dietary interaction framework may help explain poor dietary adherence and difficulties in maintaining long-term behavioral changes among patients with chronic diseases.

### 3.2 Interpersonal and social environments and social determinants

Social determinants also play an important role in psychological–dietary relationships. Socioeconomic status, food accessibility, food insecurity, educational level, health literacy, and cultural dietary norms may all affect the ability of patients with chronic diseases to obtain healthy foods, understand dietary recommendations, and maintain long-term dietary behaviors (32). Global food security data showed that approximately 2.33 billion people faced moderate or severe food insecurity in 2023, and more than 2.8 billion people could not afford a healthy diet in 2022 (33). For patients with chronic diseases, this means that even when they understand healthy dietary recommendations, they may still find it difficult to follow dietary plans over the long term because of real-life constraints.

Food insecurity and low socioeconomic status may further increase the difficulty of disease management. Taking diabetes as an example, food insecurity is not only associated with the risk of developing T2DM, but may also be related to poorer glycemic control, complications, hospitalization risk, and mental health status (34). At the same time, lower educational level and inadequate health literacy may increase the difficulty patients face in understanding complex dietary recommendations. For example, dietary management in chronic diseases often involves multiple requirements related to the intake of salt, fat, carbohydrates, protein, potassium, phosphorus, and fluids. If these recommendations are not explained adequately, they may easily lead to confusion, anxiety, and difficulties in implementation (35). In addition, diet is closely related to family relationships, traditional dietary practices, and identity. If dietary recommendations ignore patients’ cultural backgrounds, their acceptability and sustainability may be reduced (36). Therefore, dietary interventions for chronic diseases should not focus only on individual willingness, but should also consider patients’ economic conditions, food environments, health literacy, and cultural dietary backgrounds to develop dietary plans that are understandable and sustainable.

### 3.3 Food addiction and nutritional psychiatry: biological mechanisms underlying psychological–dietary relationships

Beyond the traditional framework of psychological factors, food addiction and nutritional psychiatry provide different perspectives for understanding the relationship between psychological factors and dietary behaviors. The food addiction model suggests that certain high-sugar, high-fat, and ultra-processed foods may reinforce reward responses, increase sensitivity to food cues, and weaken eating control, leading some individuals to display addiction-like behaviors such as strong food cravings, difficulty reducing intake, and repeated overeating (37). Systematic reviews and meta-analyses have shown that the weighted mean prevalence of food addiction assessed using the Yale Food Addiction Scale (YFAS) is approximately 20%, with higher proportions observed in clinical populations such as those with binge eating disorder (38). These findings suggest that highly rewarding foods may be involved in the development of emotional eating, impulsive eating, and loss of dietary control. However, it should be emphasized that food addiction remains a controversial concept. Current studies largely rely on scale-based assessments, and “food addiction” has not yet been established as a widely accepted independent clinical diagnosis. Its diagnostic boundaries, core addictive substances, neurobiological mechanisms, and overlap with emotional eating, binge eating disorder, and habitual high-energy food intake remain unclear (39). Therefore, in chronic disease management, patients’ unhealthy dietary behaviors should not be directly regarded as addiction. Rather, food addiction may serve as a useful concept for explaining highly rewarding food intake, strong food cravings, and loss of dietary control.

Nutritional psychiatry provides a broader perspective by focusing on the relationships among diet quality, nutritional status, and mental health, which may involve multiple biological processes (15, 40). Randomized controlled trials and meta-analyses suggest that improving diet quality may help reduce depressive symptoms, although the evidence for anxiety outcomes remains less consistent (41, 42). These findings support a potential role of diet in mental health, but should still be interpreted cautiously in relation to study design, population characteristics, and intervention intensity.

Overall, food addiction and nutritional psychiatry offer complementary perspectives on psychological–dietary relationships. The former focuses more on highly rewarding foods, food cravings, and loss of dietary control, whereas the latter emphasizes the broader relationship between dietary patterns, nutritional status, and mental health. Together, these perspectives suggest that dietary management in chronic diseases should consider not only nutritional intake, but also the psychological and behavioral factors that may influence eating patterns and long-term dietary management.

### 3.4 Life-course and developmental perspectives

Most existing studies have focused on adulthood, but interactions between psychology and diet may already be shaped in early life and may have certain influences on the risk of chronic diseases in adulthood. Maternal nutritional status during pregnancy, maternal psychological stress, and the family dietary environment may affect offspring neurodevelopment, appetite regulation, stress responses, and metabolic characteristics through developmental origins mechanisms (43). During childhood and adolescence, the family food environment, parental feeding practices, body image perceptions, and peer influences may also gradually shape individual dietary habits, emotion regulation patterns, and health behavior patterns (44). In addition, current evidence has relatively well demonstrated associations between early-life nutrition, maternal metabolic status during pregnancy, and the family dietary environment and subsequent obesity and cardiometabolic risk. However, there remains a lack of sufficiently long-term randomized controlled studies with clinical endpoints as primary outcomes to determine whether early nutritional or psychological interventions can improve chronic disease outcomes in adulthood (45). For example, the STRIP study provided long-term individualized dietary counseling from infancy and followed participants into young adulthood, with findings suggesting improvements in dietary quality and some cardiometabolic risk indicators (46). However, these findings mainly reflect improvements in indicators and do not demonstrate an actual reduction in the incidence of CVD or diabetes.

Therefore, from a life-course perspective, psychological–dietary interactions are not only related to current behaviors, but may also reflect psychological and behavioral patterns that develop over time. This perspective supports greater attention to early-life prevention through maternal nutritional and psychological support during pregnancy, a healthy family food environment, and the development of healthy eating behaviors and emotion regulation capacity in children. However, whether these early-life strategies ultimately reduce the risk of chronic diseases in adulthood requires further long-term evidence.

## 4 Effects of diet on psychological status

Dietary behaviors may also, in turn, influence emotion, self-evaluation, and physiological status, thereby further affecting the long-term management capacity of patients with chronic diseases. Loss of dietary control, excessive restriction, or repeated deviation from dietary plans may trigger guilt, self-blame, shame, and frustration, and may weaken patients’ confidence in continuing dietary management. Conversely, reasonable and sustainable dietary behaviors may enhance self-efficacy and a sense of control. Meanwhile, dietary patterns and nutrient intake may also affect psychological status through pathways such as inflammation and metabolism.

### 4.1 Immediate effects of eating behaviors on emotion

Eating behaviors themselves may have immediate feedback effects on emotion, thereby forming short-term dynamic cycles between diet and emotion. Regarding the effects of high-sugar, high-fat, or high-energy foods on emotion, existing evidence mainly comes from experimental studies, mood induction studies, food cue studies, and neuroimaging studies. These studies suggest that highly rewarding foods may activate reward-related pathways within a short period and provide experiences of pleasure or stress relief (47–49). However, this type of evidence mainly supports the presence of short-term emotional regulation and reward responses, and does not indicate that high-sugar and high-fat foods are beneficial for long-term mental health. If individuals rely on such foods for emotional regulation over the long term, they may subsequently experience guilt, shame, a sense of loss of dietary control, or lower mood states, which may further reinforce the cycle between negative emotions and unhealthy dietary behaviors.

Evidence on the relationship between healthy eating and positive emotions has more often come from diary studies, ecological momentary assessment (EMA), longitudinal observational studies, and some intervention studies. Diary studies and EMA can record changes in diet and emotion in daily life and are therefore well suited to capturing short-term dynamic relationships (50, 51). For example, previous diary studies have found that fruit and vegetable intake is associated with increased positive emotions on the following day (52). Smartphone-based studies in real-life settings have also suggested that healthy food choices are related to greater eating-related well-being (53). Longitudinal observational studies have further suggested that increased fruit and vegetable intake may be associated with subsequent improvements in well-being and life satisfaction (54). However, these studies may still be influenced by confounding factors such as lifestyle, socioeconomic status, physical activity, sleep, and overall health status.

By contrast, RCTs and meta-analyses of intervention studies provide stronger evidence regarding the effects of dietary interventions. The SMILES RCT showed that, among adults with moderate to severe depression, the remission rate of depression was 32.3% in the intervention group receiving 12 weeks of modified Mediterranean dietary support, compared with 8.0% in the social support control group (41). An RCT conducted among young adults also found that increasing fruit and vegetable intake improved vitality, motivation, and some indicators of psychological well-being, but did not significantly improve depressive or anxiety symptoms (55). Systematic reviews and meta-analyses generally support that higher diet quality and greater fruit and vegetable intake are associated with better mental health (56, 57). Meta-analyses of dietary interventions have also shown that dietary improvement may reduce depressive symptoms, although the evidence for improvement in anxiety symptoms is relatively weaker (42, 58). Therefore, the feedback effects of dietary behaviors on psychological status should be understood from different dimensions. The mood improvement associated with high-sugar and high-fat foods is more likely to reflect short-term reward and emotional regulation effects, whereas the influence of healthy dietary patterns on mental health is more likely to be long-term, cumulative, and shaped by individual background factors.

### 4.2 Medium-term regulatory effects of nutrients, dietary components, and biological mechanisms

Beyond immediate behavioral feedback, diet may also have relatively sustained effects on psychological status through pathways involving nutritional metabolism, neuroendocrine regulation, reward processing, immune-inflammatory responses, and the gut microbiota. For patients with chronic diseases, psychological stress, dietary behaviors, nutritional status, metabolic changes, and disease burden are often intertwined and may jointly affect emotional status, dietary adherence, and long-term self-management capacity.

At the nutrient level, omega-3 fatty acids and B vitamins are often used to explain the link between nutritional status and mental health. Omega-3 fatty acids are involved in neuronal membrane composition, inflammatory regulation, and neurotransmitter signaling (59, 60). Previous RCTs and meta-analyses have suggested that omega-3 fatty acid supplementation may have some beneficial effects on depressive symptoms, but the overall effect appears to be small and may be influenced by factors such as the composition of eicosapentaenoic acid (EPA) and docosahexaenoic acid (DHA), dosage, baseline severity of depression, and whether supplementation is used as adjunctive treatment (61, 62). B vitamins are also involved in homocysteine metabolism and monoamine neurotransmitter synthesis (63, 64), but findings from supplementation studies are similarly inconsistent. A systematic review and meta-analysis showed that B vitamin supplementation may have a small effect on reducing stress levels, but its effects on depressive and anxiety symptoms remain unclear (65). Therefore, for patients with chronic diseases, nutrient supplementation is more appropriately considered as an individualized strategy after nutritional assessment, rather than as a psychological intervention routinely applicable to all patients.

In addition, the HPA axis and reward processing may also help explain the link between dietary behaviors and emotional status under stress. Cortisol responses after stress may be involved in changes in appetite and eating behaviors (22). Long-term disease burden, repeated treatment-related stress, and dietary restrictions may also affect cortisol secretion rhythms, appetite regulation, energy intake, insulin sensitivity, and fat storage through the HPA axis (66). Meanwhile, high-sugar, high-fat, and highly processed foods may enhance the reward value of food, increase sensitivity to food cues, and provide short-term experiences of pleasure or stress relief. Neuroimaging studies suggest that individuals with higher food addiction-like scores may show stronger responses in some reward-related brain regions when exposed to highly rewarding food cues (67). However, relevant systematic reviews have also noted that findings on neural activation are not entirely consistent across studies, and most studies have been cross-sectional in design (68). Therefore, the HPA axis and reward processing may be involved in the development of food craving, emotional eating, and loss of dietary control, but unhealthy dietary behaviors should not be directly equated with addictive behaviors.

Inflammatory responses have also been proposed as one potential pathway linking diet and psychological status. Intake of high-sugar foods, high saturated fat, low dietary fiber, and highly processed foods may be associated with chronic low-grade inflammation, which may in turn affect neurotransmitter metabolism, neuroendocrine regulation, and emotional status (69–71). Phillips et al. found in an adult sample that individuals with higher dietary inflammatory potential were more likely to have depressive symptoms, anxiety, and lower psychological well-being (72). Further systematic reviews and dose–response meta-analyses have also shown that higher dietary inflammatory index scores are associated with increased risks of depression, anxiety, and psychological distress (73). The gut–brain axis provides another mechanistic framework for understanding the relationships among diet, gut microbiota, and psychological status. The gut microbiota may communicate bidirectionally with the central nervous system through short-chain fatty acids, tryptophan metabolism, immune-inflammatory responses, the vagus nerve, and neuroendocrine pathways (74). Some original studies have supported the effects of diet on the gut microbiota. For example, a high-fermented-food diet may increase gut microbiota diversity and reduce some inflammation-related markers, whereas the immune and microbial responses to a high-fiber diet may be related to an individual’s baseline microbiota status (75). However, current research on the gut–brain axis remains limited in terms of causal inference and clinical translation, and evidence on gut-targeted interventions, such as probiotics, prebiotics, dietary fiber, and fermented foods, for mental health outcomes remains inconsistent (40, 76). Therefore, the gut–brain axis is more appropriately regarded as a potential mechanism for explaining the relationship between diet and psychological status, rather than as an established clinical intervention pathway.

Overall, nutrients, the HPA axis, reward processing, inflammatory responses, and the gut–brain axis collectively suggest that diet and psychological status may be linked through multiple biological pathways. However, most of these mechanisms do not represent a single causal chain, but are jointly influenced by psychological stress, disease burden, dietary patterns, nutritional status, medication use, lifestyle, and the social environment. Therefore, in chronic disease management, biological mechanisms should be presented as possible pathways for explaining psychological–dietary interactions, rather than as mechanisms that have been fully established or can be directly translated into a unified clinical intervention strategy.

### 4.3 Long-term shaping: cumulative effects of dietary patterns on mental health

Long-term dietary patterns may be associated with mental health, and these relationships may be influenced by nutritional status, metabolic status, lifestyle, and other factors. The Mediterranean diet is one of the healthy dietary patterns that has been widely studied in the field of mental health. It is characterized by higher intake of vegetables, fruits, whole grains, legumes, nuts, olive oil, and fish, together with reduced intake of red meat, processed meat, and highly processed foods (77). The SMILES trial provided some support for the potential benefits of a Mediterranean-style dietary intervention for depressive symptoms (41). Analyses of depression outcomes from the Prevención con Dieta Mediterránea (PREDIMED) randomized trial also suggested that the Mediterranean diet, particularly the Mediterranean diet supplemented with nuts, may be associated with a lower risk of depression, although findings were not entirely consistent across different subgroups and intervention approaches (78). Overall, the Mediterranean diet may be one healthy dietary pattern that supports both chronic disease management and mental health.

The Dietary Approaches to Stop Hypertension (DASH) diet was initially developed mainly for blood pressure control and cardiometabolic risk management (79) and therefore has strong clinical applicability among patients with hypertension, CVD, and metabolic abnormalities. Some studies have also suggested that it may be relevant to mental health. For example, a randomized clinical trial among women with T2DM found improvements in scores related to depression, anxiety, stress, and sleep after a 12-week DASH dietary intervention (80). Another longitudinal study found that higher DASH and Mediterranean-DASH Intervention for Neurodegenerative Delay (MIND) diet scores were associated with a lower risk of developing depressive symptoms (81).

Plant-based and anti-inflammatory diets have also received increasing attention. The potential health effects of plant-based diets depend largely on diet quality. High-quality plant-based diets generally emphasize whole grains, vegetables, fruits, legumes, and nuts, whereas plant-based diets high in refined grains, sweets, sugar-sweetened beverages, and highly processed foods may not have the same effects. Therefore, not all plant-based diets should be assumed to be beneficial for mental health (82). Anti-inflammatory diets emphasize greater intake of vegetables, fruits, whole grains, legumes, nuts, fish, and foods rich in unsaturated fatty acids, while reducing refined carbohydrates, sugar-sweetened beverages, processed meats, and highly processed foods (83). Systematic reviews and meta-analyses suggest that higher dietary inflammatory potential is associated with increased risks of depression and anxiety (73, 84). However, the current evidence is still largely observational, and the effects of anti-inflammatory diets on psychological outcomes require further validation.

Overall, different healthy dietary patterns may be associated with mental health, although the strength of evidence is not entirely consistent across dietary approaches. In chronic disease management, the value of a dietary plan lies not only in selecting a single “optimal diet,” but also in balancing disease control, nutritional adequacy, psychological burden, and feasibility in daily life. Dietary recommendations should therefore be adjusted according to disease type, metabolic status, and individual circumstances, with greater emphasis on long-term sustainability. Healthy eating should not be reduced to a single fixed dietary pattern or overly strict dietary restrictions.

## 5 Manifestations of psychological–dietary interactions in chronic diseases

The relationship between psychological factors and dietary behaviors may vary across chronic diseases, reflecting differences in disease symptoms, treatment requirements, dietary restrictions, and the burden of long-term self-management. Difficulties in maintaining dietary management over time may increase psychological stress and the burden of behavioral maintenance, while changes in psychological status may also affect subsequent dietary management and self-management (85–87). Table 1 provides an overview of these disease-specific features, which are discussed in greater detail below.

### 5.1 Obesity

Obesity is one of the chronic diseases in which the interactions between psychological factors and dietary behaviors are particularly evident. Negative emotions and chronic stress may increase the likelihood of emotional eating and intake of energy-dense foods, and these responses may involve the HPA axis and reward system (9, 88–90). Although eating may sometimes provide short-term emotional relief, the subsequent sense of loss of control, guilt, and self-negation may further increase psychological burden and make weight management and long-term dietary maintenance more difficult.

In addition to emotional eating, weight stigma is another psychological factor that deserves attention in obesity management. Schvey et al. randomly assigned 73 women to a weight-stigmatizing video or a neutral video and found that women with overweight consumed significantly more energy after viewing the weight-stigmatizing video, suggesting that stigmatizing experiences may trigger short-term overeating rather than promote dietary control (91). Another US study reported that 42% of participants had experienced weight stigma, which was associated with disordered eating, comfort eating, sleep problems, and alcohol use (92). Longitudinal research has also shown that individuals reporting weight discrimination had a higher risk of developing obesity after 4 years, while those with obesity at baseline were also more likely to remain obese (93). Therefore, weight stigma should not be used as a strategy to promote behavior change in obesity management. Greater attention should instead be paid to its potential negative effects, including shame, self-blame, stress-related eating, and healthcare avoidance.

### 5.2 Type 2 diabetes mellitus

The long-term management of T2DM relies heavily on sustained dietary control, blood glucose monitoring, medication use, and lifestyle modification (94). In this process, it is particularly important to distinguish diabetes distress from clinical depression. Diabetes distress refers more to the disease-specific emotional burden that patients experience during long-term diabetes management, and is often related to blood glucose monitoring, dietary restrictions, medication use, hypoglycemia risk, concerns about complications, and a sense of failure in self-management. In contrast, clinical depression is a mental disorder that meets diagnostic criteria, and its effects are not limited to diabetes management itself (95, 96).

Diabetes distress is relatively common among patients with T2DM and both overlaps with and differs from depressive symptoms. The 2021 National Health Interview Survey in the United States showed that more than half of adults with diabetes had varying degrees of diabetes distress, with adjusted prevalence rates of moderate and severe diabetes distress of 24.3 and 6.6%, respectively (25). A study of Chinese adults with T2DM also reported a prevalence of moderate to severe diabetes distress of 34.64% (97). A longitudinal study by Fisher et al. among patients with T2DM found that the association between diabetes distress and HbA1c was more stable than that of clinical depression or general depressive symptoms (98). Therefore, psychological–dietary interactions in T2DM should not be simply summarized as “depression affects dietary adherence.” Greater attention should be paid to how diabetes distress, fear of hypoglycemia, and dietary guilt are embedded in daily dietary management. For patients with prominent diabetes distress, intervention priorities should include diabetes education, problem-solving training, self-management support, more flexible dietary planning, management of fear of hypoglycemia, and reduction of dietary guilt. Patients who reach the level of clinical depression should receive further psychological or psychiatric assessment.

### 5.3 Cardiovascular disease

In CVD, psychological status and dietary behaviors may be related to disease management through multiple pathways involving metabolism, inflammation, and health behaviors. Stress, anxiety, and depression may be accompanied by greater intake of high-fat, high-sugar, or high-salt foods, while long-term unhealthy dietary patterns are associated with dyslipidemia, insulin resistance, elevated blood pressure, and low-grade inflammation (7, 8, 99). In contrast, healthy dietary patterns such as the Mediterranean and DASH diets may not only support the management of cardiovascular risk factors, but may also be associated with better mental health status (77, 78, 80).

In addition to biological pathways, behavioral factors are also important in this process. Psychological stress, anxiety, and depression may make it more difficult for patients to maintain healthy diets, regular physical activity, and medication use over time, and may also affect sleep and other health behaviors (100–103). Life’s Essential 8, proposed by the American Heart Association, incorporates diet, physical activity, sleep, smoking, body weight, blood lipids, blood glucose, and blood pressure into the assessment of cardiovascular health, further highlighting the need to consider both lifestyle behaviors and physiological indicators in CVD management (104). Therefore, psychological–dietary relationships in CVD should not be understood solely through inflammatory or metabolic mechanisms, but should also take into account the potential influence of psychological status on the maintenance of health behaviors and long-term risk factor management.

### 5.4 Chronic gastrointestinal diseases

In chronic gastrointestinal diseases such as irritable bowel syndrome (IBS) and inflammatory bowel disease (IBD), psychological–dietary interactions may involve reciprocal relationships between symptom experiences and dietary behaviors. When patients experience symptoms such as abdominal pain, bloating, diarrhea, or nausea after eating, they may easily attribute these discomforts to specific foods and gradually develop food-related anxiety and dietary avoidance (105). A study by Melchior et al. involving 955 patients with IBS showed that 13.2% of patients had severe food avoidance and restriction. These patients had more severe symptoms, lower quality of life, and lower total energy, protein, and carbohydrate intake (26). Therefore, although short-term dietary avoidance may help patients gain a certain sense of control, long-term excessive avoidance may be associated with reduced food variety, inadequate nutrient intake, and poorer quality of life.

In addition, dietary management itself may also increase patients’ psychological burden. The low-FODMAP diet has some value in the management of IBS symptoms, but its implementation usually involves short-term restriction, gradual reintroduction, and individualized adjustment and should not be regarded as a fixed, long-term restrictive diet (106). Whelan et al. also noted that dietary management for IBS should not only focus on symptom improvement, but should also consider the burden, nutritional risks, and long-term sustainability associated with dietary restriction (107). A systematic scoping review found that dietary avoidance and restrictive eating behaviors are relatively common among patients with IBD and are associated with concerns about symptom recurrence, disease activity, and food safety (108). A prospective multicenter observational study by Day et al. found that poorer food-related quality of life among patients with IBD was associated with restrictive eating behaviors, disease activity, and a history of surgery (109). The study by Yelencich et al. further suggested that avoidant/restrictive eating problems deserve attention in patients with IBD, indicating that clinically significant restrictive eating may occur in this population and warrants recognition (110). Bonsack et al. also found in an IBD nutrition clinic study that food exclusion and fasting behaviors were common among patients with IBD, suggesting that clinical dietary guidance should help prevent patients from independently expanding the scope of dietary restrictions (111).

Therefore, integrated management of psychological–dietary interactions in chronic gastrointestinal diseases should not only emphasize foods to avoid, but should also address food-related anxiety, excessive avoidance, inadequate nutrition, and reduced quality of life, with the aim of improving symptom control, dietary quality, and psychological burden simultaneously.

### 5.5 Chronic kidney disease

The long-term management of CKD relies heavily on sustained and complex dietary control. Patients usually need to adjust their intake of protein, sodium, potassium, phosphorus, and fluids according to disease stage, kidney function level, and dialysis status, and these requirements may also need to be continuously modified as the disease condition changes (112). Therefore, dietary management in CKD is not only a matter of nutrient intake, but also a long-term self-management burden.

Dietary restriction fatigue is an important issue that should be emphasized in psychological–dietary interactions in CKD. A qualitative evidence synthesis by Palmer et al. on patients’ experiences of dietary and fluid restrictions in CKD found that patients commonly described these restrictions as burdensome, often involving social limitations, disruption of habits, repeated resistance to temptation, and the need to balance health goals with daily life needs (27). An integrative review by Lambert et al. on dietary adherence in end-stage kidney disease reported an overall renal diet adherence rate of approximately 31.5%, highlighting the challenges of maintaining complex dietary recommendations over time (113). Therefore, dietary nonadherence among patients with CKD should not be simply interpreted as a lack of cooperation, but should be considered in the context of long-term dietary restrictions, complex information, psychological stress, and real-life circumstances.

In real-world nephrology settings, however, integrated psychological–dietary management still faces implementation barriers. Nephrology outpatient clinics usually need to address kidney function, biochemical indicators, blood pressure, anemia, dialysis preparation, and medication adjustment within a limited consultation time, and psychological screening and dietary behavior assessment may easily be placed in a secondary position. Many healthcare institutions also lack dietitians and mental health professionals who can be involved in long-term CKD management. Even when dietary restriction fatigue, depression, anxiety, or severe dietary nonadherence is identified, continuous referral and follow-up pathways may not always be available. Therefore, difficulties in dietary management among patients with CKD are often not a single adherence problem, but rather the result of complex dietary restrictions, long-term psychological burden, and related factors.

## 6 Interventions and clinical applications: developing an integrated psychological–dietary management model

The preceding sections have discussed the potential bidirectional relationship between psychological factors and dietary behaviors in chronic disease management. In clinical practice, a more important question is how these interactions can be incorporated into long-term management. Integrated psychological–dietary management is not simply a combination of nutritional and psychological interventions. Rather, it involves addressing patients’ psychological burden, dietary behaviors, and adherence within routine care, with assessment, stratified intervention, referral, and continued follow-up provided according to individual needs. At present, this model is better regarded as an integrative approach based on existing evidence from nutritional, psychological, and behavioral interventions, and its overall clinical effectiveness still requires further evaluation.

### 6.1 Differences between integrated psychological–dietary management and traditional multidisciplinary management

Traditional multidisciplinary chronic disease management usually emphasizes the joint participation of physicians, nurses, dietitians, mental health professionals, and other healthcare providers in disease management. Its advantage is that patients’ physiological indicators, dietary plans, physical activity behaviors, and psychological problems can be addressed from different professional perspectives. However, in real-world implementation, this model may sometimes still take the form of parallel involvement by multiple professionals, in which different professionals provide separate recommendations and patients are expected to understand and integrate them on their own. For patients with poor long-term adherence, heavy psychological burden, or complex dietary restrictions, excessive and fragmented recommendations may instead increase confusion and implementation burden.

The framework proposed here places greater emphasis on addressing psychological status and dietary behaviors simultaneously during chronic disease management. Its focus is not to simply add nutritional advice to psychological support, but to understand patients’ emotional status, dietary implementation, disease indicators, and capacity for behavioral maintenance within the same management process during screening, intervention, and follow-up. This approach may help identify the psychological stress, implementation difficulties, and real-life contextual limitations underlying poor dietary adherence, and may provide a basis for subsequent stratified interventions.

### 6.2 Screening, comprehensive assessment, and risk stratification

Integrated psychological–dietary management first requires an overall assessment of the patient. In addition to routine disease-related indicators, attention should be given to psychological status, dietary behaviors, readiness for behavior change, self-efficacy, and social support. Psychological assessment may include depression, anxiety, stress, disease-related distress, and quality of life, while dietary assessment may consider dietary patterns, dietary adherence, emotional eating, and the burden of dietary management. For patients with marked weight concerns, binge eating, excessive dieting, or other abnormal eating behaviors, the potential risk of eating disorders should also be considered.

The focus of assessment may differ across diseases. In patients with obesity, greater attention may be given to emotional eating, loss-of-control eating, and weight stigma. For patients with T2DM, diabetes distress and fear of hypoglycemia may be particularly relevant. In patients with IBS and IBD, eating-related anxiety, food avoidance, and excessive restriction should be considered. For patients with CKD, dietary restriction fatigue and difficulties in understanding and following complex dietary recommendations may deserve greater attention. Such assessments may help identify the psychological and behavioral barriers that patients experience during long-term dietary management.

On this basis, preliminary stratification may be considered according to psychological burden, dietary behavior problems, and the difficulty of disease management to help determine subsequent management priorities. Patients with relatively mild psychological burden and stable dietary management may continue to receive routine nutrition education, individualized dietary guidance, and self-monitoring. For those with mild to moderate anxiety, depression, stress, emotional eating, or reduced adherence, behavioral support, cognitive behavioral strategies, mindful eating, and regular follow-up may be added to nutritional guidance. Patients with marked depression, severe loss-of-control eating, binge eating, risk of eating disorders, persistent treatment nonadherence, or risk of self-harm should be referred promptly to psychological or psychiatric specialists, with clinicians, dietitians, and mental health professionals involved in subsequent management. This stratification is intended mainly to help identify different management needs, and its specific criteria and clinical applicability require further validation.

### 6.3 Individualized nutritional intervention and the practical positioning of precision nutrition

Individualized nutritional intervention remains a foundation of chronic disease management. Its goal is not only to improve disease-related indicators, but also to help patients maintain appropriate dietary patterns over time. In practice, dietary plans should be adjusted according to disease type, disease stage, nutritional status, psychological burden, and patients’ life circumstances. For patients with T2DM, the structure and timing of carbohydrate intake and the prevention of hypoglycemia may be prioritized (114). For patients with CVD, the focus may be placed on adjusting fatty acid composition, sodium intake, and overall dietary quality (115). Patients with IBS may follow a low-FODMAP diet under professional guidance, while long-term excessive restriction should be avoided (116). Patients with CKD need to adjust protein, sodium, potassium, phosphorus, and fluid intake according to kidney function, serum potassium and phosphorus levels, dialysis status, and nutritional risk (117). The effectiveness of nutritional intervention also depends on whether dietary recommendations can be maintained in daily life. Overly strict or insufficiently explained dietary restrictions may increase the risks of inadequate nutrition, dietary anxiety, reduced quality of life, and poor adherence (85, 116). Dietary recommendations should therefore allow sufficient flexibility. Dietitians should not only provide dietary plans, but also help patients understand dietary goals, identify practical barriers, and adjust recommendations according to patients’ psychological status and life circumstances.

In recent years, individualized nutritional intervention has gradually moved toward precision nutrition. More targeted nutritional strategies may in the future be informed by indicators such as inflammatory status, gut microbiota characteristics, metabolomics, and tryptophan metabolism (118). However, the clinical application of precision nutrition in integrated psychological–dietary management remains at an early stage. Its implementation may be limited by the high costs of multi-omics testing and continuous monitoring, as well as challenges in data collection and interpretation (119). Excessive reliance on genetic, microbiome, or metabolomic testing may also overlook patients’ economic circumstances, food accessibility, cultural dietary habits, and psychological and behavioral barriers (120). At present, a more feasible approach is to provide stratified and individualized nutritional guidance based on routine dietary assessment, disease-related indicators, psychological status, and life circumstances, rather than routinely applying high-cost testing to all patients.

### 6.4 Psychological and behavioral interventions: CBT, mindful eating, and self-management support

Psychological and behavioral interventions aim to improve patients’ emotion regulation, cognitive appraisal, and behavioral control. Cognitive behavioral therapy (CBT) is one of the most widely used psychological interventions. Its core purpose is to help patients identify and modify negative cognitions related to disease, body shape, food, and self-management, thereby improving emotional responses and promoting healthy behaviors (121). Within the proposed integrated framework, CBT may be incorporated to address negative cognitions in dietary management, emotional eating, a sense of dietary failure, and reduced adherence. Relevant studies suggest that CBT may alleviate symptoms of anxiety and depression (122–124), and may also improve patients’ adherence to dietary and lifestyle recommendations (125). However, the implementation of CBT in chronic disease management still faces barriers related to accessibility, cost, and resource availability. Full CBT usually requires trained psychotherapists and multiple sessions, which may not be easily accessible for patients with multiple chronic diseases, limited mobility, heavy financial burden, or residence in areas with insufficient healthcare resources (126). In recent years, internet-based or mobile CBT has shown certain effects among patients with chronic diseases, but differences remain across disease types, platform formats, guidance intensity, and patient adherence (127).

Similarly, mindful eating and other mindfulness-based interventions may serve as adjunctive strategies in integrated psychological–dietary management. Mindfulness training emphasizes awareness and acceptance of present-moment experiences, which may help patients distinguish physiological hunger from emotional eating and habitual eating, while reducing guilt and self-blame after eating (128, 129). Systematic reviews and meta-analyses suggest that mindfulness-related interventions may help improve some eating behaviors, such as emotional eating, binge eating, or mindless eating, but their effects on physiological indicators such as body weight, blood pressure, and blood glucose are not consistent (130, 131). Therefore, mindful eating is more suitable for improving eating awareness, reducing dietary guilt, and alleviating emotional eating, rather than being regarded as a core intervention that can independently replace nutritional therapy, psychotherapy, or pharmacological treatment.

Behavioral interventions are an important way to translate health-related cognition into long-term behavior change. Goal setting, self-monitoring, and staged feedback are usually more helpful for maintaining dietary behavior change than health education alone. Studies have shown that, in the management of T2DM (132) and hypertension (133), patient-centered self-management support may improve dietary control behaviors and some physiological indicators. The key lies in helping patients maintain relatively stable healthy dietary behaviors and disease self-management even under conditions such as stress and negative emotions.

### 6.5 Digital monitoring, long-term follow-up, and implementation pathways

Digital tools can be used to record changes in diet, mood, physical activity, sleep, and disease indicators, providing supportive assistance for long-term follow-up and individualized feedback. Among these tools, ecological momentary assessment (EMA) may serve both as a research tool and as a potential clinical monitoring tool (134, 135). As a research tool, EMA can be used to capture short-term dynamic relationships among psychological status, dietary impulses, eating behaviors, and symptom changes in patients’ natural living environments, thereby reducing recall bias and revealing within-person changes. As a clinical tool, EMA may be used for continuous monitoring of diet, mood, sleep, symptoms, and disease indicators in high-risk patients, providing a reference for individualized feedback and follow-up adjustment (136, 137). However, EMA is currently not suitable as a routine monitoring method for all patients with chronic diseases. Its clinical application remains limited by factors such as patient recording burden, insufficient long-term adherence, missing data, privacy protection, and healthcare professionals’ workload. Therefore, EMA is more appropriate as a research method and an auxiliary monitoring tool for some high-risk patients, rather than as a replacement for routine clinical assessment. For healthcare institutions with limited resources, brief questionnaires, dietary records, follow-up interviews, and basic digital tools may be prioritized for screening and follow-up, while more complex digital monitoring can be reserved for patients at high risk or those requiring more refined management.

In addition, long-term follow-up should address both physiological indicators and behavioral and psychological outcomes. In addition to disease indicators such as body weight, blood glucose, blood pressure, blood lipids, and kidney function, assessments should also include dietary adherence, psychological distress, self-efficacy, quality of life, and patients’ evaluation of the sustainability of dietary plans. Continuous follow-up and dynamic adjustment may facilitate the incorporation of psychological and dietary care into long-term chronic disease management, rather than limiting care to one-time health education or a single session of dietary guidance.

The implementation of integrated psychological–dietary management may vary across healthcare settings. In institutions with sufficient professional resources, clinicians, dietitians, and mental health professionals may jointly participate in patient assessment and intervention. In primary care or resource-limited settings, brief psychological and dietary screening, basic guidance, and routine follow-up may be provided first, with further referral considered according to individual needs. In practice, factors such as consultation time, availability of trained professionals, and referral pathways may affect implementation. Therefore, specific management processes should be adapted to local healthcare resources and patient needs.

## 7 Current limitations and future directions

Existing studies provide an important basis for understanding the relationship between psychological factors and dietary behaviors, but several limitations remain. A considerable proportion of the current evidence comes from cross-sectional and observational studies, making it difficult to determine temporal sequence and causal direction. Psychological status and dietary behaviors may influence each other during long-term chronic disease management, while both may also be affected by factors such as socioeconomic status, food accessibility, education, and physical activity. If these factors are not adequately controlled, the observed associations may be overestimated or underestimated.

Differences across study populations also limit the generalizability of current findings. Although obesity, T2DM, CVD, chronic gastrointestinal diseases, and CKD may all involve psychological and dietary issues, their main psychological burdens, dietary restrictions, and behavioral barriers are not the same. Age, sex, cultural dietary background, socioeconomic conditions, and disease stage may also influence how these relationships are expressed and the feasibility of interventions. Findings from a particular disease group or population should therefore not be directly generalized to all patients with chronic diseases.

Current studies also have limitations in measurement methods. Psychological status and dietary behaviors are still commonly assessed using self-report scales, dietary questionnaires, or retrospective reports, which are susceptible to recall bias, social desirability bias, and reporting errors. These methods may also have difficulty capturing short-term changes in emotion, eating behavior, and disease symptoms. EMA, digital dietary records, and wearable devices may provide more continuous information, but they also face challenges related to recording burden, missing data, privacy, and cost. The available evidence may also be affected by publication bias, as studies with positive findings are generally more likely to be published. In addition, as this is a narrative review, standardized literature selection and quality assessment procedures used in systematic reviews were not applied. Therefore, some selection bias may have occurred during literature selection and evidence interpretation, and some relevant studies may not have been included.

Future research should include more longitudinal studies, RCTs, and mechanistic studies to further clarify the temporal sequence and potential pathways linking psychological status, dietary behaviors, and chronic disease outcomes. The applicability of this framework should also be examined across different disease stages, cultural dietary backgrounds, and socioeconomic settings, with greater attention to negative or inconsistent findings. In addition to traditional clinical outcomes, future studies should consider dietary adherence, psychological distress, quality of life, patient acceptability, cost-effectiveness, and long-term maintenance. Integrated psychological–dietary management still requires stronger evidence and more real-world studies to further clarify its clinical value.

## 8 Conclusion

Existing evidence suggests that psychological status and dietary behaviors may be bidirectionally related during chronic disease management. These relationships may involve emotional responses, behavioral adherence, biological mechanisms, and disease burden, although their specific manifestations differ across chronic diseases. Psychological screening, individualized nutritional guidance, behavioral support, digital monitoring, and multidisciplinary collaboration have some supporting evidence and may provide complementary support for long-term chronic disease management.

Building on this evidence, this review further proposes an integrated psychological–dietary management framework that brings psychological assessment, dietary management, and behavioral support into a common management approach. However, this framework is currently based mainly on the integration of existing evidence, and its overall clinical effectiveness, target populations, and specific implementation strategies have not yet been fully established. Current evidence also remains limited by insufficient causal inference, study heterogeneity, and limited real-world implementation data.

Therefore, before integrated psychological–dietary management can be further developed into a routine or standardized approach for chronic disease management, more prospective studies, RCTs, and real-world implementation studies are needed to further evaluate its effectiveness, feasibility, and implementation pathways.
