# Supplementary material for: Psychological–dietary interactions in chronic disease management: mechanisms and integrated intervention strategies
Source: Front Nutr. 2026 Sep 9;13:1925415. doi: 10.3389/fnut.2026.1925415 (PMC13598965; doi:10.3389/fnut.2026.1925415)
Supplement: Supplementary file 1 [file Table_1.docx]

Table S1 Detailed database-specific search strategies for literature identification

| **Database** | **Search fields/approach** | **Search strategy** | **Search period** |
| --- | --- | --- | --- |
| PubMed | MeSH terms and Title/Abstract | ("Stress, Psychological"[Mesh] OR "Anxiety"[Mesh] OR "Anxiety Disorders"[Mesh] OR "Depression"[Mesh] OR "Depressive Disorder"[Mesh] OR "Psychological Distress"[Mesh] OR "Self Efficacy"[Mesh] OR stress[Title/Abstract] OR "psychological stress"[Title/Abstract] OR "perceived stress"[Title/Abstract] OR "emotional stress"[Title/Abstract] OR anxiety[Title/Abstract] OR depression[Title/Abstract] OR depressive[Title/Abstract] OR "psychological distress"[Title/Abstract] OR "emotional distress"[Title/Abstract] OR "disease-related distress"[Title/Abstract] OR "disease related distress"[Title/Abstract] OR "self-efficacy"[Title/Abstract] OR "self efficacy"[Title/Abstract]) AND ("Diet"[Mesh] OR "Feeding Behavior"[Mesh] OR "Diet Therapy"[Mesh] OR diet[Title/Abstract] OR dietary[Title/Abstract] OR "dietary behavior"[Title/Abstract] OR "dietary behaviour"[Title/Abstract] OR "eating behavior"[Title/Abstract] OR "eating behaviour"[Title/Abstract] OR "emotional eating"[Title/Abstract] OR "dietary adherence"[Title/Abstract] OR "dietary compliance"[Title/Abstract] OR "dietary restriction"[Title/Abstract] OR "dietary restrictions"[Title/Abstract]) AND ("Obesity"[Mesh] OR "Diabetes Mellitus, Type 2"[Mesh] OR "Cardiovascular Diseases"[Mesh] OR "Gastrointestinal Diseases"[Mesh] OR "Renal Insufficiency, Chronic"[Mesh] OR obesity[Title/Abstract] OR "type 2 diabetes mellitus"[Title/Abstract] OR "type 2 diabetes"[Title/Abstract] OR T2DM[Title/Abstract] OR "cardiovascular disease"[Title/Abstract] OR "cardiovascular diseases"[Title/Abstract] OR "gastrointestinal disease"[Title/Abstract] OR "gastrointestinal diseases"[Title/Abstract] OR "gastrointestinal disorder"[Title/Abstract] OR "gastrointestinal disorders"[Title/Abstract] OR "irritable bowel syndrome"[Title/Abstract] OR IBS[Title/Abstract] OR "inflammatory bowel disease"[Title/Abstract] OR "inflammatory bowel diseases"[Title/Abstract] OR IBD[Title/Abstract] OR "chronic kidney disease"[Title/Abstract] OR "chronic kidney diseases"[Title/Abstract] OR CKD[Title/Abstract]) | From inception to April 2026 |
| Web of Science | Topic (TS) | TS=(("psychological stress" OR "perceived stress" OR "emotional stress" OR anxiety OR anxious OR depression OR depressive OR "psychological distress" OR "emotional distress" OR "disease-related distress" OR "disease related distress" OR "self-efficacy" OR "self efficacy") AND (diet OR dietary OR "dietary behavior" OR "dietary behaviors" OR "dietary behaviour" OR "dietary behaviours" OR "eating behavior" OR "eating behaviors" OR "eating behaviour" OR "eating behaviours" OR "emotional eating" OR "dietary adherence" OR "dietary compliance" OR "dietary restriction" OR "dietary restrictions") AND (obesity OR obese OR "type 2 diabetes mellitus" OR "type 2 diabetes" OR "type II diabetes mellitus" OR "type II diabetes" OR T2DM OR T2D OR "cardiovascular disease" OR "cardiovascular diseases" OR "gastrointestinal disease" OR "gastrointestinal diseases" OR "gastrointestinal disorder" OR "gastrointestinal disorders" OR "irritable bowel syndrome" OR IBS OR "inflammatory bowel disease" OR "inflammatory bowel diseases" OR IBD OR "chronic kidney disease" OR "chronic kidney diseases" OR CKD)) | From inception to April 2026 |
| Embase | Emtree terms and Title/Abstract/Author keywords | (('mental stress'/exp OR 'anxiety'/exp OR 'anxiety disorder'/exp OR 'depression'/exp OR 'psychological distress'/exp OR 'self efficacy'/exp OR 'psychological stress':ti,ab,kw OR 'mental stress':ti,ab,kw OR 'perceived stress':ti,ab,kw OR 'emotional stress':ti,ab,kw OR anxiety:ti,ab,kw OR anxious:ti,ab,kw OR depression:ti,ab,kw OR depressive:ti,ab,kw OR 'psychological distress':ti,ab,kw OR 'emotional distress':ti,ab,kw OR 'disease-related distress':ti,ab,kw OR 'disease related distress':ti,ab,kw OR 'self-efficacy':ti,ab,kw OR 'self efficacy':ti,ab,kw) AND ('diet'/exp OR 'feeding behavior'/exp OR 'diet therapy'/exp OR 'diet restriction'/exp OR diet:ti,ab,kw OR dietary:ti,ab,kw OR 'dietary behavior':ti,ab,kw OR 'dietary behaviors':ti,ab,kw OR 'dietary behaviour':ti,ab,kw OR 'dietary behaviours':ti,ab,kw OR 'eating behavior':ti,ab,kw OR 'eating behaviors':ti,ab,kw OR 'eating behaviour':ti,ab,kw OR 'eating behaviours':ti,ab,kw OR 'emotional eating':ti,ab,kw OR 'dietary adherence':ti,ab,kw OR 'dietary compliance':ti,ab,kw OR 'dietary restriction':ti,ab,kw OR 'dietary restrictions':ti,ab,kw) AND ('obesity'/exp OR 'non insulin dependent diabetes mellitus'/exp OR 'cardiovascular disease'/exp OR 'gastrointestinal disease'/exp OR 'irritable colon'/exp OR 'inflammatory bowel disease'/exp OR 'chronic kidney failure'/exp OR obes*:ti,ab,kw OR 'type 2 diabetes mellitus':ti,ab,kw OR 'type 2 diabetes':ti,ab,kw OR 'type II diabetes mellitus':ti,ab,kw OR 'type II diabetes':ti,ab,kw OR 'non insulin dependent diabetes mellitus':ti,ab,kw OR T2DM:ti,ab,kw OR T2D:ti,ab,kw OR NIDDM:ti,ab,kw OR 'cardiovascular disease':ti,ab,kw OR 'cardiovascular diseases':ti,ab,kw OR 'gastrointestinal disease':ti,ab,kw OR 'gastrointestinal diseases':ti,ab,kw OR 'gastrointestinal disorder':ti,ab,kw OR 'gastrointestinal disorders':ti,ab,kw OR 'irritable bowel syndrome':ti,ab,kw OR IBS:ti,ab,kw OR 'inflammatory bowel disease':ti,ab,kw OR 'inflammatory bowel diseases':ti,ab,kw OR IBD:ti,ab,kw OR 'chronic kidney disease':ti,ab,kw OR 'chronic kidney diseases':ti,ab,kw OR 'chronic renal disease':ti,ab,kw OR 'chronic renal failure':ti,ab,kw OR CKD:ti,ab,kw)) | From inception to April 2026 |
| Scopus | Title/Abstract/Keywords | TITLE-ABS-KEY(("psychological stress" OR "perceived stress" OR "emotional stress" OR anxiety OR anxious OR depression OR depressive OR "psychological distress" OR "emotional distress" OR "disease-related distress" OR "self-efficacy") AND (diet OR dietary OR "dietary behavior" OR "eating behavior" OR "emotional eating" OR "dietary adherence" OR "dietary compliance" OR "dietary restriction") AND (obesity OR obese OR "type 2 diabetes mellitus" OR "type 2 diabetes" OR "type II diabetes mellitus" OR "type II diabetes" OR T2DM OR T2D OR "cardiovascular disease" OR "gastrointestinal disease" OR "gastrointestinal disorder" OR "irritable bowel syndrome" OR IBS OR "inflammatory bowel disease" OR IBD OR "chronic kidney disease" OR "chronic renal disease" OR "chronic renal failure" OR CKD)) | From inception to April 2026 |
